# Age-dependent evaluation of organ and effective doses in pediatric full-spine radiography: influence of anteroposterior and posteroanterior projection and copper filtration using Monte Carlo simulation

**DOI:** 10.1007/s00247-025-06452-7

**Published:** 2025-12-11

**Authors:** Yasushi Katsunuma, Kaoru Sato

**Affiliations:** 1 Department of Radiology, Tokai University Hospital, Isehara, Kanagawa, Japan; 2 Nuclear Science and Engineering Center, Nuclear Science Research Institute, Japan Atomic Energy Agency, Tokai, Ibaraki, Japan

**Keywords:** Pediatric scoliosis, Spine radiography, Radiation dose, Monte Carlo method, Copper filtration

## Abstract

**Background:**

Repeated full-spine radiography for scoliosis follow-up in children results in increased radiation exposure, especially to anterior radiosensitive organs. Optimizing projection direction and beam filtration is essential for dose reduction.

**Objective:**

To quantitatively evaluate the age-dependent effects of anteroposterior (AP) and posteroanterior (PA) projections, with and without a 0.1-mm copper filter, on organ and effective doses in pediatric full-spine radiography.

**Materials and methods:**

Monte Carlo simulations were performed using the Particle and Heavy Ion Transport code System with 5-, 10-, and 15-year-old female hybrid phantoms. Full-spine radiography from the first cervical vertebra to both femoral heads was modeled under AP and PA conditions, with or without copper filtration. Organ doses were calculated, with active bone marrow and bone surface evaluated using the “International Commission on Radiological Protection Publication 116” dose response functions. Percentage depth dose analysis was performed to assess the effect of body thickness.

**Results:**

PA projection markedly reduced doses to anterior radiosensitive organs, with maximum reductions of approximately 93% for the breast (AP/PA ratio 14) and over 80% for the thyroid. Copper filtration provided additional reductions of 15–19% in AP and 5–6% in PA. In contrast, dose increases were observed in posterior and deep-seated organs such as the kidneys and active bone marrow. Effective dose was reduced by about half with PA and further decreased with copper filtration.

**Conclusion:**

PA projection and copper filtration are effective strategies for reducing radiation exposure to anterior radiosensitive organs and lowering effective dose in pediatric full-spine radiography. However, dose increases in deep-seated organs were also observed, highlighting the need for protocol optimization according to patient age and organ location.

**Graphical Abstract:**

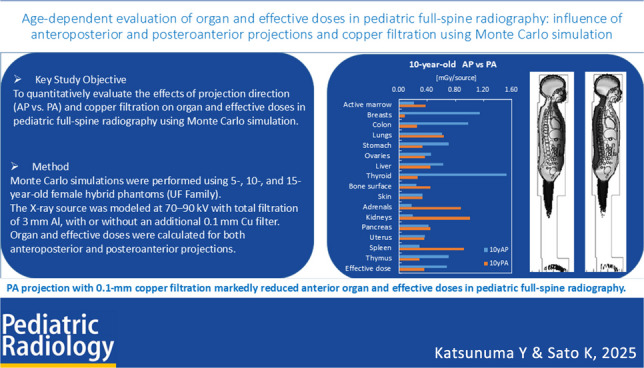

**Supplementary Information:**

The online version contains supplementary material available at 10.1007/s00247-025-06452-7.

## Introduction

Adolescent idiopathic scoliosis is a common condition during the growth period, particularly in girls, with a female-to-male ratio reaching as high as 10:1 in advanced cases [1]. Diagnosis and follow-up of adolescent idiopathic scoliosis typically require repeated full-spine radiographs, with one study reporting an average of 22 examinations over 3 years and a cumulative effective dose of approximately 20 mSv [2]. Such repeated exposures raise concerns about long-term effects on radiosensitive organs such as the breast and thyroid. Large-scale epidemiological studies have also suggested an association between pediatric X-ray exposure and an increased risk of breast cancer [3, 4]. From the perspective of pediatric radiation protection, dose reduction in adolescent idiopathic scoliosis patients is therefore an important international priority.

Changing the projection direction is one of the most effective strategies for radiation dose reduction. Monte Carlo analyses and corresponding clinical measurements have indicated that switching from anteroposterior (AP) to posteroanterior (PA) projection in lumbar spine radiography can reduce the effective dose by up to 53% and decrease breast dose to less than one-third compared with AP projection [2]. Furthermore, comparative studies between the EOS low-dose biplanar imaging system (EOS Imaging, Paris, France) and conventional digital radiography have also demonstrated that PA projection achieves greater dose reduction while maintaining diagnostic image quality [5, 6]. In addition, a clinical study by Minehiro et al. [7] showed that adding a 0.1-mm copper filter in pediatric full-spine radiography for adolescent idiopathic scoliosis patients reduced organ doses by approximately 20–40% and the effective dose by about 30%, while preserving image quality. Reflecting these findings, the Society on Scoliosis Orthopaedic and Rehabilitation Treatment (SOSORT) guidelines recommend PA projection in adolescent idiopathic scoliosis patients [8].

In addition, age and body size can significantly affect radiation dose. International surveys have reported age-dependent differences in pediatric organ doses of up to two- to threefold [9], highlighting the need for age-stratified evaluation that considers changes in body thickness and organ depth with growth. In clinical pediatric radiography, tube voltage is generally adjusted according to age: around 70 kVp for 5-year-olds, 80 kVp for 10-year-olds, and 90 kVp for 15-year-olds [7, 10–12]. Diagnostic X-ray equipment is required to provide a minimum total filtration of 2.5 mm aluminum at tube voltages exceeding 70 kVp [13]. The 3.0-mm aluminum filter used in this study effectively removed unnecessary low-energy photons, thereby reducing the skin dose while maintaining diagnostic image quality [14–16].

The use of additional filtration has also been recognized as effective for dose reduction. A 0.1-mm copper filter has been widely reported as a practical means to achieve both dose reduction and preservation of image quality in pediatric imaging [17, 18], and its effectiveness has also been demonstrated in adolescent idiopathic scoliosis patients [19]. However, most previous studies have focused on younger pediatric phantoms [17, 20], and age-dependent effects considering growth-related changes in body thickness and organ position have not been sufficiently investigated. In particular, differences in AP/PA projection may vary with body thickness, and percentage depth dose analysis [21] is a useful tool for understanding this aspect.

The purpose of this study was to quantitatively evaluate, using Monte Carlo simulation, the age-dependent effects of projection direction (AP vs. PA) and the use of copper filtration on organ and effective doses in pediatric full-spine radiography. In addition, by incorporating percentage depth dose analysis, we aimed to clarify the physical effects of growth-related changes in body thickness on dose distribution, and to comprehensively evaluate the effectiveness and limitations of the PA combined with copper filtration approach. Unlike previous studies, our work provides a systematic age-stratified analysis across multiple pediatric phantoms and combines Monte Carlo dose estimation with percentage depth dose evaluation, offering new insights into dose optimization for scoliosis imaging.

## Methods

### Phantom and irradiation setup

**Fig. 1:**
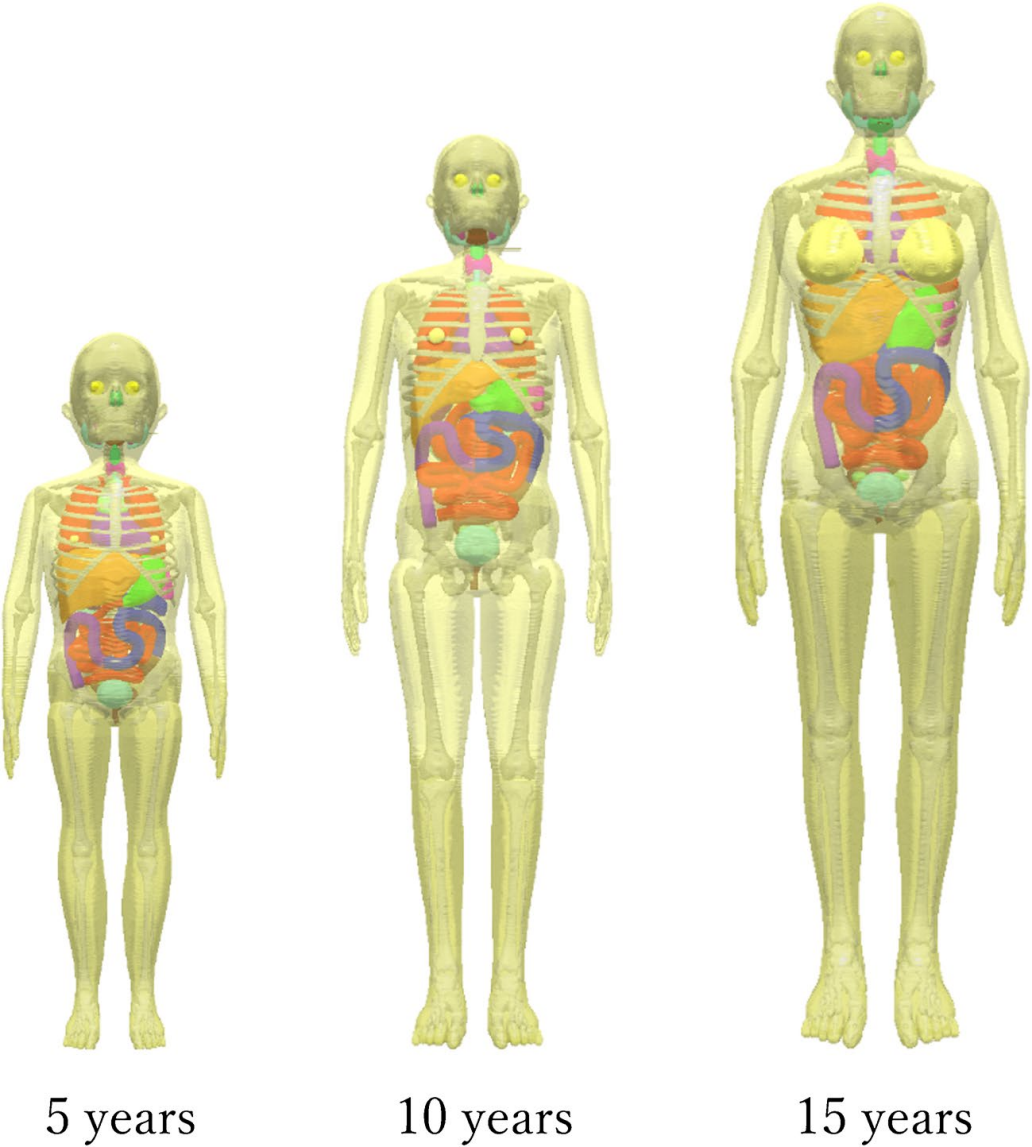
University of Florida family hybrid voxel phantoms of 5-year-old, 10-year-old, and 15-year-old females. These models were used to evaluate age-dependent variations in organ and effective doses in full-spine radiography

This study employed the University of Florida family hybrid voxel phantoms (5-year-old, 10-year-old, and 15-year-old female models) developed by the University of Florida [22] (Fig. 1). These phantoms reproduce 37 organs and tissues defined in “International Commission on Radiological Protection Publications 89” [23] and “International Commission on Radiological Protection Publications 110” [24] with a voxel resolution of 1 mm^3^, accurately reflecting growth-related changes in body size and organ position in children. Physical properties of bone and soft tissue were assigned according to the densities and elemental compositions specified in “International Commission on Radiation Units and Measurements Publications 44” [25] and “International Commission on Radiological Protection Publications 46” [26]. Because this study aimed to evaluate radiation doses to sex-specific organs such as the breast and ovaries, female models were used for all age groups.

**Fig. 2:**
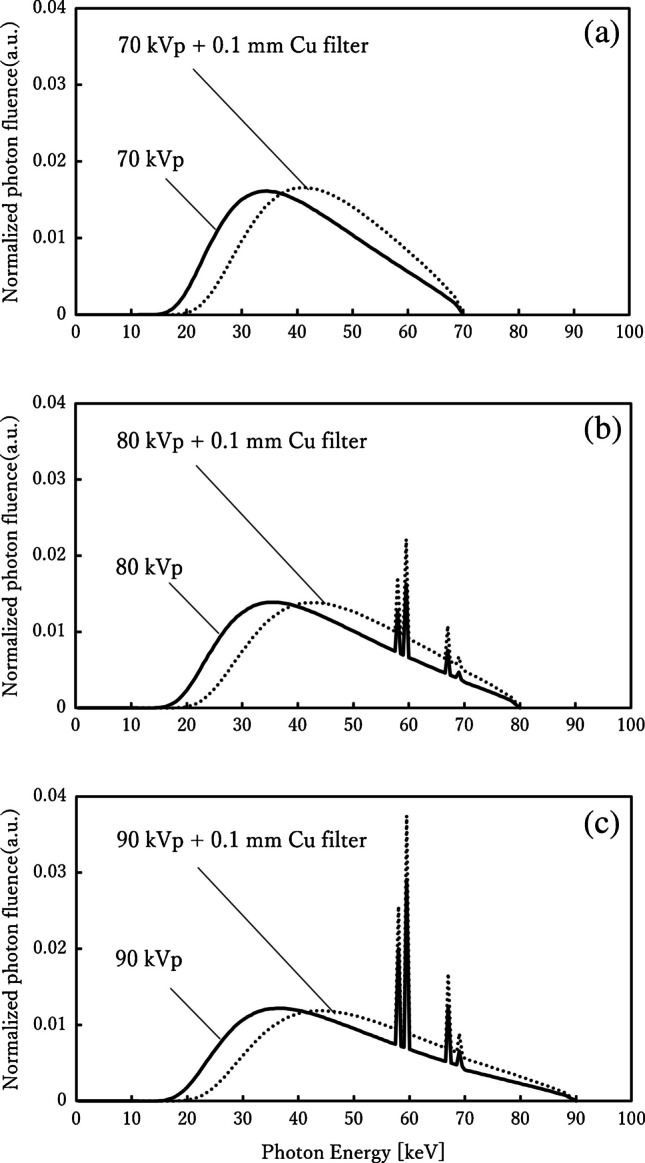
Incident X-ray spectra generated using SpekCalc at tube voltages of 70 kVp, 80 kVp, and 90 kVp with 3.0-mm aluminum filtration and a 12° target angle. Spectra are shown in three panels: (**a**) 70 kVp, (**b**) 80 kVp, and (**c**) 90 kVp, each comparing conditions with and without a 0.1-mm copper (Cu) filter. The corresponding first half-value layers (HVLs) and effective energies were as follows: 70 kVp – 2.63 mm Al/31.8 keV (without Cu) and 4.00 mm Al/44.8 keV (with Cu); 80 kVp – 3.00 mm Al/33.5 keV (without Cu) and 4.57 mm Al/48.7 keV (with Cu); 90 kVp – 3.38 mm Al/35.2 keV (without Cu), and 5.14 mm Al/52.2 keV (with Cu). *Solid lines* represent spectra without the copper filter, and *dotted lines* represent those with it, clearly demonstrating the beam-hardening effect of copper filtration resulting in increased HVL and effective energy. *Cu*, copper; *HVL*, half-value layer

X-ray energy spectra used for irradiation were then generated using SpekCalc version 1.00 (Institute of Cancer Research, London, UK) [27, 28] with tube voltages of 70 kVp, 80 kVp, and 90 kVp. The total filtration was set to 3.0 mm aluminum with a target angle of 12°, and the resulting spectra were converted into the input format of the Particle and Heavy Ion Transport code System (Japan Atomic Energy Agency, Tokai, Japan) input format. For comparison, spectra with and without a 0.1-mm copper filter are also presented in Fig. 2, while the detailed modeling of copper filtration is described in the following section. Two projection conditions were modeled: AP and PA. The source-to-phantom distance (SPD), defined as the distance from the X-ray source to the mid-plane of the phantom, was fixed at 180 cm, with the beam centered on the seventh thoracic vertebra. The irradiation field was set as a rectangle covering from the first cervical vertebra (C1) to both proximal femoral heads, consistent with clinical protocols for scoliosis radiography. Similar field designs have been adopted in previous studies of adolescent idiopathic scoliosis patients, reflecting the clinical requirement to include the entire spine during follow-up examinations, although variations in both the upper and lower borders exist among institutions [2, 7, 14, 20]. The rectangular field was generated by defining the source as a conical emission from the focal spot and applying a physical collimator immediately after emission. To ensure that this rectangular field was correctly implemented in the Particle and Heavy Ion Transport code System, the field design was verified using T-Track tally visualization, and representative results for the AP projection in 5-year-old, 10-year-old, and 15-year-old phantoms are provided in Supplementary Material 1.

To further evaluate beam hardening and dose-reduction effects, copper filtration was modeled as an additional condition.

Copper filtration has been clinically investigated as a dose-reduction strategy, particularly in pediatric radiography and scoliosis imaging, although its adoption is heterogeneous across institutions. Previous reports and international dose-optimization initiatives have demonstrated its potential to reduce patient dose while maintaining diagnostic image quality [6, 7, 17, 18, 29]. In this study, a 0.1-mm copper filter was placed in front of the X-ray source model, and the filter was explicitly modeled in the Particle and Heavy Ion Transport code System to accurately reproduce conditions with and without copper filtration. Four exposure settings were evaluated, combining projection direction (AP or PA) with the presence or absence of the copper filter (Cu ±). Dose calculations were independently performed for each phantom (5-year-old, 10-year-old, and 15-year-old).

### Monte Carlo simulation

Radiation transport calculations were performed using the general-purpose Monte Carlo particle transport code Particle and Heavy Ion Transport code System, version 3.33 [30]. The cutoff energies were set to 1 keV for photons and 10 keV for electrons, and both scattered photons and secondary electrons were included in the simulation. The number of particle histories was determined to keep the statistical uncertainty of energy deposition in each organ and tissue below 5% for all conditions. The required histories varied depending on phantom age and the use of copper filtration.

Organ and tissue doses were calculated using the T-Deposit tally in the Particle and Heavy Ion Transport code System for 37 organs and tissues defined in International Commission on Radiological Protection Publication 103 [31]. Because the red bone marrow and bone surface cannot be anatomically distinguished in the phantom, their doses were assessed following the methodology of International Commission on Radiological Protection Publication 116 [32]. Absorbed doses were first calculated from photon fluence data obtained using the T-Track tally. Dose response functions for 15 skeletal sites for active bone marrow and 32 skeletal sites for bone surface were then applied to these absorbed doses, and the results were integrated across sites in post-processing to derive the final dose estimates.

Following the framework of International Commission on Radiological Protection Publication 103, doses for 37 organs and tissues were consolidated into 27 categories where appropriate, and effective dose was calculated by applying tissue weighting factors. All results are reported per source: organ doses in mGy/source and effective dose in mSv/source.

For presentation and discussion, doses for 16 representative organs and tissues were selected from the 27 categories: the superficial organs (skin, thyroid, breast, thymus), intermediate organs (lungs, liver, stomach, colon), deep-seated organs (kidneys, adrenal glands, pancreas, spleen), skeletal/hematopoietic system (active bone marrow, bone surface), and reproductive organs (ovaries, uterus). Detailed numerical values for all 27 organs and tissues are provided in Supplementary Material 2.

In addition, representative cross-sectional dose distribution maps obtained under the Cu– condition are presented to visually illustrate spatial patterns of dose deposition within the body.

### Dose calculation and normalization

Equivalent dose (*H*_T_) for each organ was calculated by multiplying the absorbed dose (*D*_T_) by a radiation weighting factor (*ω*_R_ = 1.0). Effective dose (*E*) was then determined according to International Commission on Radiological Protection Publication 103 [31] using the tissue weighting factors (*ω*_T_):

$$E=\sum_T\omega_T\cdot H_T=\sum_T\omega_T\cdot D_T$$

Comparisons of organ and effective doses were performed using relative values. The percentage relative change was defined as:

$$Relative\,\mathrm{chang}e\;(\%)=\frac{D_{test}-D_{ref}}{D_{ref}}\times\;100$$

Where *D*_ref_ represents the reference condition, defined as AP projection without copper filtration (AP, Cu–), and *D*_test_ represents the comparison conditions: AP with copper (AP, Cu+), PA without copper (PA, Cu–), or PA with copper (PA, Cu+). Hereafter, Cu+ and Cu– denote conditions with and without a 0.1-mm copper filter, respectively.

For selected results, fold changes were also derived from relative changes to facilitate intuitive interpretation, defined as:

$$Fold\operatorname{change}=1+\left(\frac{Relative\operatorname{change}(\%)}{100}\right)$$

For example, a relative change of +231.5% corresponds to a fold change of 1+2.315=3.3-fold.

To enable direct comparison among irradiation conditions, dose normalization was subsequently performed so that the transmitted photon fluence was equivalent across conditions.

In clinical radiography, automatic exposure control (AEC) adjusts the tube current–time product (mAs) according to the transmitted dose. To simulate this behavior, dose normalization was applied using the number of directly transmitted photons (excluding scattered photons) measured at the exit surface of the phantom.

Four spherical scoring regions (diameter 1 cm each) were placed at the phantom exit surface.

Using the T-Point tally in the Particle and Heavy Ion Transport code System, the number of directly transmitted photons (excluding scattered photons) was obtained for each sphere.

For all conditions, including those with and without the copper filtration, organ and effective doses were corrected so that the transmitted photon fluence at the exit surface was equivalent.

For AP and PA projections, no additional normalization was performed because the same source conditions (i.e., the same number of emitted photons) were applied. Under conditions with identical source-to-phantom distance (SPD) and phantom geometry, it was assumed that clinical automatic exposure control (AEC) would select the same mAs regardless of projection direction. In contrast, comparisons between conditions with and without copper filtration required normalization, as the presence of the copper filter reduces the number of transmitted photons. In these cases, doses were corrected so that the number of directly transmitted photons at the exit surface was equivalent.

This normalization approach allowed the effects of copper filtration to be evaluated under equivalent image-forming photon fluence, enabling quantitative assessment of beam-hardening effects in isolation.

#### Percentage depth dose analysis

To evaluate the attenuation characteristics of X-rays within the body, homogeneous soft-tissue phantoms with a density of 1.06 g/cm^3^ were constructed according to “International Commission on Radiation Units and Measurements Publication 44” [25]. The phantom diameters were determined with reference to the median effective thoracic diameters reported in “International Commission on Radiological Protection Publications 89 [33] and 143” [34], and were set to 14 cm for the 5-year-old model, 18 cm for the 10-year-old model, and 22 cm for the 15-year-old model.

Tube voltages were assigned according to age: 70 kVp for the 5-year-old phantom, 80 kVp for the 10-year-old phantom, and 90 kVp for the 15-year-old phantom. Each condition was analyzed with and without a 0.1-mm copper filter, resulting in six simulation settings. The source-to-phantom distance (SPD) was fixed at 180 cm, and the cutoff energies in the Particle and Heavy Ion Transport code System were set to 1 keV for photons and 10 keV for electrons.

Dose calculations were performed using the T-Deposit tally in the Particle and Heavy Ion Transport code System, and absorbed dose values were obtained at 1-mm intervals along the beam depth. For Cu– conditions, the entrance surface dose (0 cm) was normalized to 100%, and percentage depth dose curves were generated for each phantom thickness (5-year-old, 0–14 cm; 10-year-old, 0–18 cm; and 15-year-old, 0–22 cm). For Cu+ conditions, where entrance dose was reduced by the filter, normalization was performed using the exit surface dose as the reference. This normalization approach enabled pure evaluation of the beam hardening effects of copper filtration on depth-dose distributions under conditions where transmitted photon fluence was equivalent.

## Results

### Absorbed dose distribution and age dependence

**Fig. 3:**
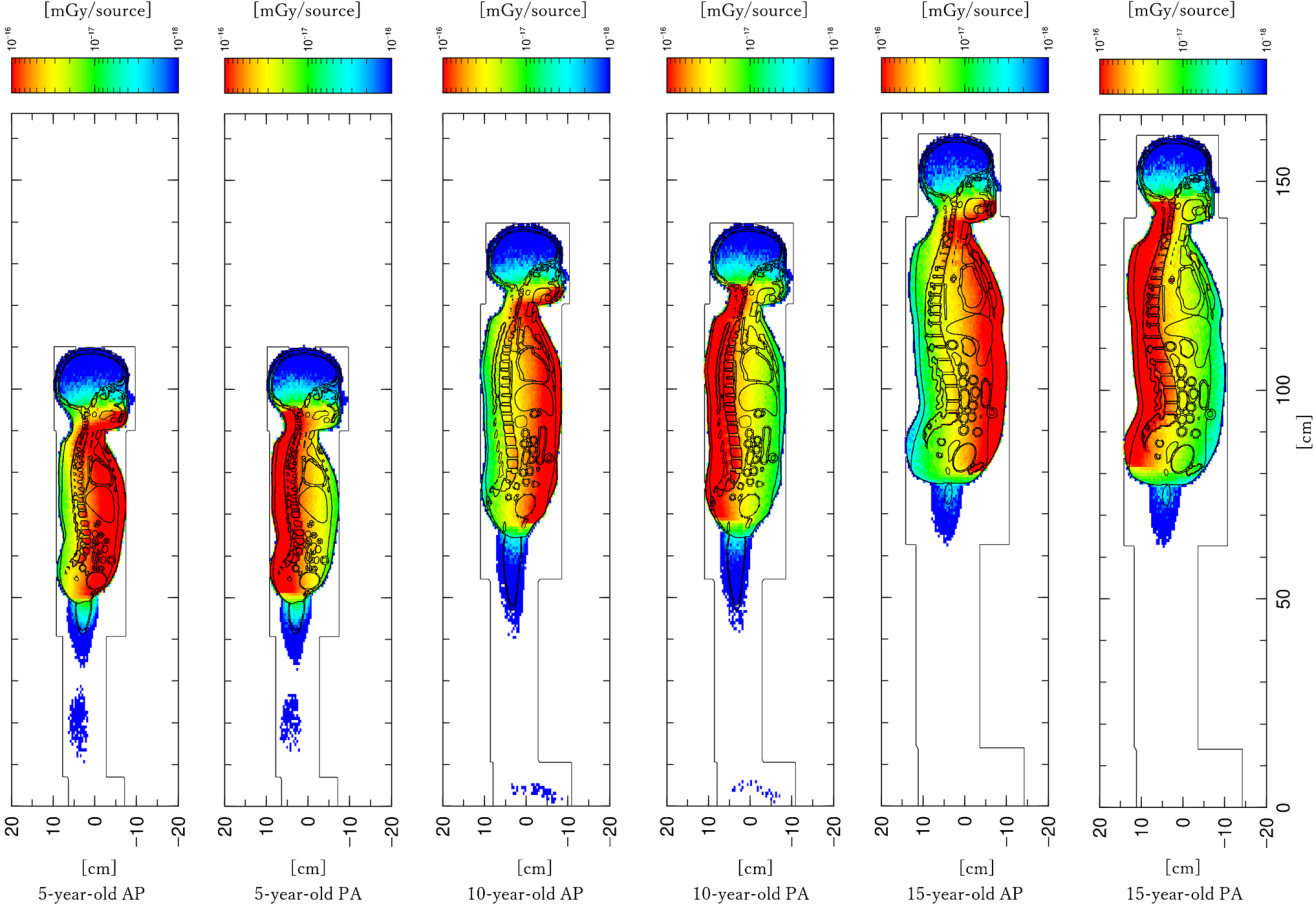
Absorbed dose distribution maps (deposit maps) in 5-year-old, 10-year-old, and 15-year-old female phantoms under anteroposterior and posteroanterior projections. Representative results are shown for conditions without copper filtration. *AP*, anteroposterior; *PA*, posteroanterior

Figure 3 shows whole-body absorbed dose distributions (deposit maps) for the 5-year-old, 10-year-old, and 15-year-old phantoms under AP and PA projections. For clarity, representative examples without copper filtration (Cu–) are presented. In all conditions, maximum absorbed doses were located on the entrance side of the X-ray beam and decreased gradually with depth, showing clear age-dependent variation in dose distribution. In younger phantoms, smaller body thickness resulted in the dose peak being located closer to the center of the trunk. In the 15-year-old phantom, the absorbed dose was more concentrated near the entrance surface, with marked attenuation in superficial regions. Thus, increasing body thickness influenced the internal dose gradient, shifting the high-dose region toward the surface.

### Differences by projection direction and out-of-field exposure

Under AP projection, higher doses were concentrated in anterior organs such as the breasts and abdominal viscera, whereas PA projection produced higher dose regions in posterior structures such as the spine and paraspinal soft tissues. In the pelvic region, AP projection yielded higher doses to the bladder, uterus, and ovaries, while PA projection resulted in higher doses to the sacrum and posterior iliac bones.

Although the irradiation field was defined from C1 to both proximal femoral heads (F1), absorbed doses were also observed outside this range, including the head and lower pelvic regions. These findings indicate that projection direction influenced not only in-field organ doses but also the distribution of out-of-field scattered radiation.

### Normalization and effect of copper filtration

For each irradiation condition, correction factors were derived to normalize dose data for copper-filter conditions, based on the number of directly transmitted photons (excluding scattered components) measured at the phantom exit surface. Table 1 presents representative photon counts and the corresponding normalization factors for each age group and tube voltage setting. The addition of the copper filter reduced the number of directly transmitted photons at the exit surface by approximately 15–25%. This reduction was smaller at higher tube voltages, and the transmitted photon count also varied with age-related differences in body thickness, demonstrating that copper filtration influenced beam quality in a manner dependent on phantom thickness and tube potential.

**Table 1:** Photon counts at the phantom exit surface and normalization factors for AP projection with and without copper filtration at each tube voltage (per source)

Tube voltage (kVp)	Age (years)	Photon counts (Cu–) (photons/source)	Photon counts (Cu+) (photons/source)	Normalization factor
70	5	6.33E-05	4.71E-05	0.74
80	10	3.76E-05	2.92E-05	0.78
90	15	1.09E-05	9.29E-06	0.85

*AP*, anteroposterior; *Cu*+, with copper filter applied; *Cu–*, without copper filter

#### Organ- and age-specific variations in absorbed dose and the effect of copper filtration

The following results are based on normalized dose values. For each age group (5-year-old, 10-year-old, and 15-year-old), the influence of projection direction (AP vs. PA) and the presence or absence of copper filtration was analyzed. In this section, organs were categorized according to anatomical location into superficial, intermediate, deep-seated, skeletal/hematopoietic, and reproductive groups. Trends in dose variation and the age dependence of copper filtration effects are summarized for each category. Normalized organ doses and effective dose are presented in Table 2, and dose reduction rates relative to AP projection without copper are shown in Table 3.

**Table 2:** Normalized organ doses (mGy/source) and effective doses (mSv/source) in 5-year-old, 10-year-old, and 15-year-old female phantoms under AP and PA projections with and without copper filtration

Organ dose (mGy/source)	5 years				10 years				15 years			
	AP	PA	AP Cu+	PA Cu+	AP	PA	AP Cu+	PA Cu+	AP	PA	AP Cu+	PA Cu+
Skin	0.54	0.53	0.38	0.38	0.33	0.33	0.25	0.25	0.33	0.32	0.24	0.23
Thyroid	2.23	0.47	1.65	0.45	1.53	0.26	1.17	0.25	1.40	0.24	1.06	0.18
Breasts	1.72	0.12	1.26	0.12	1.15	0.08	0.86	0.08	1.07	0.08	0.80	0.07
Thymus	1.46	0.40	1.19	0.37	0.70	0.29	0.64	0.28	0.74	0.21	0.62	0.20
Lungs	1.05	1.05	0.87	0.90	0.61	0.64	0.54	0.58	0.67	0.47	0.56	0.36
Liver	0.95	0.66	0.83	0.59	0.63	0.45	0.57	0.41	0.73	0.30	0.62	0.24
Stomach	1.01	0.58	0.88	0.53	0.71	0.33	0.63	0.32	0.76	0.28	0.64	0.22
Colon	1.42	0.51	1.12	0.46	0.98	0.26	0.81	0.25	0.97	0.19	0.78	0.15
Kidneys	0.40	1.34	0.39	1.13	0.20	1.01	0.20	0.87	0.17	1.00	0.16	0.84
Adrenals	0.27	1.28	0.26	1.08	0.17	0.88	0.18	0.79	0.19	0.70	0.18	0.60
Pancreas	0.65	0.78	0.59	0.71	0.42	0.44	0.40	0.42	0.34	0.47	0.31	0.43
Spleen	0.46	1.37	0.43	1.14	0.29	0.92	0.28	0.79	0.37	0.76	0.34	0.64
Red bone marrow	0.28	0.40	0.25	0.36	0.22	0.38	0.20	0.34	0.26	0.35	0.23	0.32
Bone surface	0.43	0.65	0.39	0.58	0.25	0.44	0.24	0.41	0.26	0.33	0.23	0.30
Ovaries	0.90	0.58	0.79	0.54	0.45	0.37	0.42	0.37	0.42	0.23	0.39	0.18
Uterus	0.67	0.54	0.59	0.49	0.37	0.36	0.34	0.34	0.27	0.26	0.25	0.24
Effective dose	1.05	0.55	0.85	0.50	0.67	0.36	0.57	0.33	0.67	0.28	0.55	0.26

Sixteen representative organs and the effective dose are shown; complete data for all 27 organs are provided in Supplementary Material 3

*AP*, anteroposterior; *PA*, posteroanterior; *Cu* +, with copper filter applied; *Cu–*, without copper filter

**Table 3:** Percentage reduction of organ doses and effective dose relative to the reference condition (anteroposterior projection without copper filtration, AP–Cu–). Values were calculated as the percentage difference compared with AP–Cu– to demonstrate the reduction effect of PA projection and copper filtration

	5 years				10 years				15 years			
	AP	PA	AP Cu +	PA Cu +	AP	PA	AP Cu +	PA Cu +	AP	PA	AP Cu +	PA Cu +
Skin	-	−1.5	−28.9	−29.9	-	−0.7	−25.6	−26.0	-	−2.0	−27.6	−29.0
Thyroid	-	−78.9	−26.1	−79.8	-	−82.8	−23.5	−83.4	-	−82.9	−24.1	−86.8
Breasts	-	−92.9	−26.7	−92.9	-	−93.0	−25.1	−92.7	-	−92.8	−25.4	−93.1
Thymus	-	−72.7	−18.7	−74.5	-	−59.1	−8.4	−59.7	-	−71.6	−16.2	−73.4
Lungs	-	0.0	−17.2	−14.7	-	5.0	−11.0	−5.2	-	−30.1	−16.3	−46.7
Liver	-	−30.2	−12.9	−37.3	-	−29.5	−10.1	−35.0	-	−58.4	−14.4	−67.2
Stomach	-	−42.4	−12.9	−47.5	-	−52.8	−10.2	−54.6	-	−63.8	−15.3	−71.2
Colon	-	−64.4	−20.7	−67.8	-	−73.7	−17.2	−74.7	-	−80.7	−19.1	−84.6
Kidneys	-	231.5	−4.5	179.6	-	410.6	−0.7	340.6	-	506.6	−4.8	409.2
Adrenals	-	374.0	−2.9	300.1	-	402.7	2.0	350.2	-	273.3	−4.8	219.7
Pancreas	-	19.8	−9.1	9.7	-	6.0	−2.8	0.8	-	39.2	−7.7	25.6
Spleen	-	201.0	−6.3	150.6	-	219.3	−3.3	175.3	-	103.4	−10.0	72.3
Red bone marrow	-	46.7	−11.1	29.0	-	74.5	−7.2	58.8	-	36.2	−10.9	22.1
Bone surface	-	50.9	−9.1	35.1	-	78.4	−4.9	65.3	-	29.2	−9.7	17.0
Ovaries	-	−36.0	−12.4	−39.7	-	−18.6	−6.9	−18.6	-	−46.9	−8.4	−58.6
Uterus	-	−19.6	−12.1	−26.7	-	−1.7	−8.1	−7.0	-	−5.7	−9.0	−11.2
Effective dose	-	−47.1	−19.0	−52.7	-	−47.1	−15.5	−50.6	-	−57.6	−17.9	−61.6

Sixteen representative organs and the effective dose are shown; complete data for all 27 organs are provided in Supplementary Material 3

*AP*, anteroposterior; *PA*, posteroanterior; *Cu* +, with copper filter applied; *Cu–*, without copper filter

#### Superficial organs (the skin, thyroid, breasts, thymus)

Superficial organs analyzed included the skin, thyroid, breasts, and thymus. Although all are located near the body surface, dose variation patterns differed among organs.

For the skin, which always represents the entrance surface, differences between AP and PA projections were minimal, with maximum changes of only about 2%. In contrast, copper filtration had a clear impact, reducing skin dose by approximately 25–29% under AP projection and 26–30% under PA projection, indicating that copper filtration contributed more to dose reduction than projection direction.

For the thyroid, a strong directional dependence was observed. Switching from AP to PA projection reduced dose by more than 80% across all age groups. With copper filtration, reductions reached up to approximately 87% under PA projection, confirming that both projection direction and beam quality contributed to dose reduction.

For the breasts, the largest differences were observed. In the 15-year-old phantom, dose decreased from 1.72 mGy/source with AP projection to 0.12 mGy/source with PA projection, a 93% reduction (AP/PA ratio approximately 14). Similar trends were observed in the 5-year-old and 10-year-old models, demonstrating consistent reductions across age groups. With copper filtration, an additional ~25% reduction was achieved under AP projection and a smaller reduction under PA projection, with the PA+Cu condition yielding as low as 0.09 mGy/source in the 5-year-old phantom.

For the thymus, which is also located near the body surface, a strong directional dependence was observed. The PA projection reduced the dose by 72.7%, 59.1%, and 71.6% for the 5-year-old, 10-year-old, and 15-year-old phantoms, respectively. With copper filtration, additional reductions of approximately 8–19% were achieved, with the greatest effect observed in the 5-year-old phantom.

Overall, the thyroid, breasts, and thymus showed pronounced reductions under PA projection, further enhanced by copper filtration. In contrast, the skin showed minimal directional differences, but substantial superficial dose reduction with copper. Notably, breast dose was reduced by about 93% with PA projection, quantitatively confirming the effectiveness of the PA+Cu strategy for protecting radiosensitive organs.

#### Intermediate organs (the lungs, liver, stomach, colon)

Intermediate organs analyzed included the lungs, liver, stomach, and colon. These organs are located centrally or slightly anterior within the trunk, and therefore exhibited moderate dose reductions with PA projection, though with organ-specific differences.

For the lungs, characteristic patterns were observed. In the 5-year-old phantom, no difference was found between AP and PA projections (approximately 0%), while in the 10-year-old phantom, a slight increase was observed (+5.0%). In contrast, the 15-year-old phantom showed a clear reduction (30.1%). With copper filtration, additional reductions were achieved across all ages (17.2% at 5 years, 11.0% at 10 years, and 16.3% at 15 years), reaching 46.7% in the 15-year-old phantom under PA+Cu conditions.

For the liver, consistent reductions were observed with PA projection: 30.2% at 5 years, 29.5% at 10 years, and 58.4% at 15 years. With copper filtration, further reductions of approximately 10–15% under AP and 5–10% under PA were obtained, resulting in a maximum reduction of 67.2% in the 15-year-old phantom under PA projection.

For the stomach, directional dependence was more pronounced, with reductions of 42.4% at 5 years, 52.8% at 10 years, and 63.8% at 15 years, showing a clear age-related increase in reduction.

For the colon, even greater reductions were observed: 64.4% at 5 years, 73.7% at 10 years, and 80.7% at 15 years. Under PA+Cu conditions, the 15-year-old phantom reached a maximum reduction of 84.6%, representing the largest effect among the intermediate organs evaluated.

Overall, except for the lungs, intermediate organs showed clear reductions with PA projection, further enhanced by copper filtration. The liver, stomach, and colon demonstrated an age-dependent trend, with greater reductions at older ages, reaching over 85% in the colon under PA+Cu projection. In contrast, the lungs showed an age-specific behavior, with negligible differences in younger phantoms and a significant reduction only in the 15-year-old phantom, distinguishing them from the other intermediate organs.

#### Deep-seated organs (the kidneys, adrenal glands, pancreas, spleen)

Deep-seated organs evaluated included the kidneys, adrenal glands, pancreas, and spleen. Located in the retroperitoneal region, these organs showed trends opposite to those of superficial and intermediate organs, with increased doses under PA projection. In the kidneys, PA projection resulted in increases of up to approximately sixfold (+506.6% in the 15-year-old phantom), and approximately fivefold increases were also observed in the adrenal glands. The pancreas and spleen likewise demonstrated elevated doses under PA projection across all ages, with the effect particularly pronounced in the younger phantoms.

Although the addition of copper filtration provided modest reductions of several percent to about 10%, it did not fundamentally mitigate the increased doses observed under PA projection. Overall, dose increases with PA projection were consistently observed in deep-seated organs, with the kidneys and adrenal glands showing increases ranging from +270% to +500%, clearly indicating that retroperitoneal organs receive substantially higher doses under PA projection.

#### Skeletal and hematopoietic tissues (the active bone marrow, bone surface)

Because active bone marrow and bone surface are largely distributed in posterior skeletal structures, both consistently showed increased doses under PA projection. Although the magnitude varied by age, dose increases reached approximately 1.5- to twofold for both tissues, in contrast to the trends observed for superficial and intermediate organs.

With the addition of copper filtration, reductions of about 5–11% were generally observed under AP projection; however, under PA projection, copper filtration did not provide sufficient mitigation, and the tendency toward increased dose persisted.

Overall, skeletal tissues exhibited unavoidable dose increases with PA projection, and the benefit of copper filtration remained limited. The increased dose to hematopoietic tissues represents a clinically relevant concern, highlighting a trade-off between the protection of superficial organs and the increased risk to skeletal structures.

#### Reproductive organs (the ovaries, uterus)

Because all phantoms used in this study were female models, the ovaries and uterus were evaluated as representative reproductive organs. Both are located in the anterior pelvis, anatomically positioned to receive direct exposure under AP projection.

For the ovaries, switching to PA projection reduced dose across all age groups, with decreases of approximately 36% at 5 years and 47% at 15 years. With the addition of copper filtration, further reductions were observed, reaching a maximum of about 59% at 15 years.

For the uterus, dose reductions with PA projection were also observed but were more limited: approximately 20% at 5 years and only a few percent at 10 years and 15 years. With copper filtration, additional reductions remained modest, around 5–7%.

Overall, reproductive organs were more heavily exposed under AP projection, but the combination of PA projection and copper filtration provided measurable reductions. A clear reduction was demonstrated for the ovaries, while the effect on the uterus was limited. Considering the long-term reproductive risks in pediatric patients, the PA+Cu strategy may represent an important protective approach.

#### Effective dose

Analysis of effective dose showed consistent reductions with PA projection compared with AP projection across all age groups. Relative decreases with PA projection were approximately 47% for the 5-year-old and 10-year-old phantoms and 58% for the 15-year-old phantom, indicating a trend toward greater reductions with increasing age.

With the addition of copper filtration, further decreases were observed: 15–19% for AP projection and an additional 5–6% for PA projection relative to PA without copper. Thus, the combination of PA projection and copper filtration provided the greatest dose reduction overall, confirming its quantitative effectiveness.

#### Percentage depth dose analysis

**Fig. 4:**
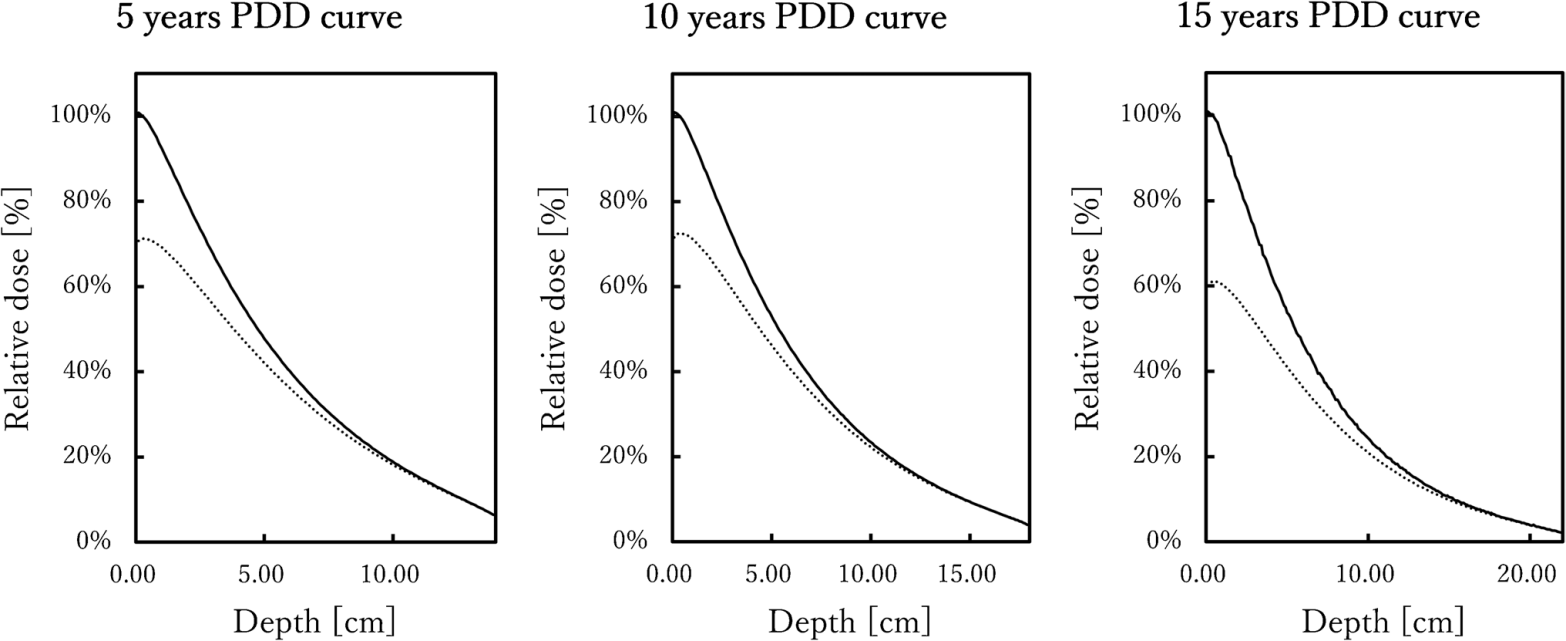
Percentage depth-dose curves for homogeneous phantoms representing 5-year-old, 10-year-old, and 15-year-old body sizes. Comparisons are shown between conditions with and without a 0.1-mm copper filter, showing marked dose reduction near the surface and minimal differences in deeper regions. *Cu*, copper

Figure 4 shows the percentage depth dose curves obtained from homogeneous soft-tissue phantoms of different diameters corresponding to ages 5  years, 10  years, and 15 years, with and without copper filtration.

In the superficial region (0–10% of body thickness), a steep dose falloff was observed across all age groups, and the addition of copper filtration markedly reduced entrance surface dose.

In the intermediate region (30–50% of body thickness), the 5-year-old phantom showed the highest relative dose, followed by the 10-year-old and 15-year-old phantoms. The dose reduction effect of copper filtration was smaller than in the superficial region, and the differences between curves diminished in this depth range.

In the deep region (≥70% of body thickness), the influence of copper filtration became minimal, and the percentage depth dose curves converged. In contrast, age-dependent differences were evident, with higher ages showing more gradual attenuation and consequently higher relative doses at greater depths.

In this study, Cu– conditions were normalized to the entrance surface dose (0 cm), whereas Cu+ conditions were normalized to the exit surface dose. This approach ensured equivalence of transmitted photon fluence, allowing a direct comparison of the isolated beam-hardening effect of copper filtration on depth-dose distributions.

#### Validation

To confirm the validity of the Monte Carlo dose evaluation model developed in this study, comparisons were made with previously reported clinical measurements and Monte Carlo analyses.

In the 15-year-old phantom, breast dose was 1.72 mGy/source under AP projection and 0.12 mGy/source under PA projection, corresponding to an AP/PA ratio of 14.3. A prior study reported breast doses of 0.77 mGy (AP) and 0.07 mGy (PA), with an AP/PA ratio of approximately 11 [35], showing good agreement with our findings. Furthermore, previous reports noted a 90–95% reduction in breast dose with PA projection [6], which was consistent with the ~93% reduction observed in this study.

Regarding copper filtration, dose reductions of 25.4% (anteroposterior) and 25.7% (posteroanterior) were observed in this study, aligning well with previously reported reductions of approximately 25–30% in pediatric phantom Monte Carlo simulations under similar conditions [20]. In addition, comparisons of organ dose between Monte Carlo simulation and thermoluminescent dosimeter measurements have demonstrated good agreement, with discrepancies typically within approximately 10–15% [36], and in our study, differences for breast dose remained within approximately 13%. Taken together, the results obtained in this study are quantitatively consistent with multiple independent reports, supporting the validity and reproducibility of the simulation model.

## Discussion

In this study, 5-year-old, 10-year-old, and 15-year-old female hybrid phantoms were used to quantitatively evaluate, by means of Monte Carlo simulation, the effects of projection direction (AP vs. PA) and the presence or absence of copper filtration on organ and effective doses in pediatric full-spine radiography. In addition, percentage depth dose analysis was incorporated to elucidate the physical background of dose distribution in relation to beam quality, body thickness, and projection direction.

The findings of this study demonstrated depth-dependent trends in dose variation according to the anatomical location of each organ. Superficial organs showed marked dose reductions and intermediate organs exhibited moderate changes, whereas deep-seated organs and skeletal tissues tended to show dose increases. In the following discussion, organ dose characteristics are described sequentially according to anatomical depth.

First, for superficial organs such as the skin, thyroid, breasts, and thymus, high doses were observed under AP projection because these structures are directly exposed to the incident X-ray beam, whereas switching to PA projection substantially reduced the dose. For example, in the 15-year-old phantom, breast dose decreased from 1.72 mGy/source under AP projection to 0.12 mGy/source with PA projection, representing a reduction of approximately 93% (AP-to-PA ratio approximately 14). This trend was consistently observed across all age groups. Similarly, in the thyroid, dose reduction reached about 87% under PA projection with copper filtration. Percentage depth dose analysis confirmed that low- to medium-energy photons contributed significantly to superficial dose deposition, and that the removal of soft X-rays by copper filtration effectively protected anterior organs.

Clinically, the breast and thyroid are among the most radiosensitive organs in children, and dose reduction is directly linked to lowering long-term cancer risk. In the follow-up of adolescent idiopathic scoliosis, full-spine radiography is typically performed about 20 times from adolescence onward, and cumulative exposure to the breast and thyroid critically influences future risk. The present findings quantitatively demonstrate that PA projection combined with copper filtration provides effective protection for anterior radiosensitive organs.

In contrast, for intermediate organs such as the lungs, liver, stomach, and colon, neither the pronounced dose reductions observed in superficial organs nor the increases seen in deep-seated organs were evident. Instead, an intermediate behavior was demonstrated. The lungs and liver showed moderate dose reductions of approximately 10–30% with PA projection, whereas the colon exhibited only small differences between AP and PA projections, with the influence of copper filtration remaining limited. These organs are centrally located within the trunk, where attenuation occurs through anterior tissues under AP projection and through posterior tissues under PA projection. As a result, partial dose mitigation occurs under both directions. This was consistent with percentage depth dose analysis, which showed relatively small differences between AP and PA in the intermediate depth range.

Clinically, although dose variations in intermediate organs are less striking than in superficial organs, their contribution to cumulative dose from repeated examinations is not negligible. In particular, the liver and small intestine are moderately radiosensitive, and due to their tissue weighting factors, they substantially affect effective dose. Therefore, protocol optimization should consider not only the protection of highly radiosensitive organs such as the breast and thyroid but also the cumulative dose to intermediate organs. The present findings quantitatively demonstrated that PA projection combined with copper filtration provided measurable reductions even for intermediate organs, supporting their role in comprehensive dose optimization.

For deep-seated organs such as the kidneys, adrenal glands, pancreas, spleen, and other retroperitoneal structures, dose increases were clearly observed with PA projection. For the kidneys and adrenal glands, PA+Cu projection resulted in doses several times higher than those under AP projection, and similar trends were seen in the spleen. This increase can be attributed to beam hardening, in which removal of low-energy components by copper filtration produces a harder spectrum, allowing higher-energy X-rays to penetrate more deeply. Percentage depth dose analysis also demonstrated that high-energy components were retained in the deeper regions, consistent with the finding of relatively higher deep-organ doses under PA projection. The kidneys and adrenal glands are critical organs that must remain functional throughout childhood and beyond. Although their radiosensitivity is lower than that of superficial organs, the potential impact of cumulative radiation exposure on renal function and tumor risk cannot be disregarded. Thus, these findings highlight the need to balance the benefit of superficial organ protection with the potential increase in deep-organ dose when selecting PA projection.

The present study quantitatively clarified the impact of PA projection and copper filtration on deep-organ dose, demonstrating the existence of this trade-off.

Additionally, skeletal and hematopoietic tissues, including active bone marrow and bone surface, exhibited dose increases of approximately 75% and 78%, respectively, under PA+Cu projection. These tissues are widely distributed in posterior structures such as the spine and pelvis, making them more directly exposed under PA projection. This was consistent with percentage depth dose analysis, which demonstrated retention of dose at greater depths.

Active bone marrow serves as the central site of hematopoiesis during childhood, and its increased dose may be associated with long-term risks such as radiation-induced leukemia. The International Commission on Radiological Protection assigns specific tissue weighting factors to both active bone marrow and bone surface, meaning that dose increases in these tissues contribute directly to a higher effective dose and carry important clinical implications.

Therefore, when selecting PA projection, the protective benefit for anterior radiosensitive organs must be weighed against the increased risk of exposure to skeletal and hematopoietic tissues. The present study quantitatively demonstrated that PA projection leads to increased doses in these tissues.

Regarding reproductive organs, the ovaries and uterus showed distinct depth-dependent behavior. Because these organs are located in the anterior pelvis, they received higher doses under AP projection due to direct beam exposure. The ovaries, situated at a depth of approximately 10–30% of body thickness, showed substantial reductions with PA projection as they were displaced from the entrance field. In contrast, the uterus, located deeper (about 30–50% of body thickness), showed only limited differences between AP and PA projections, consistent with percentage depth dose analysis, and exhibited smaller dose reductions compared with the ovaries. The addition of copper filtration provided further reductions for both organs, with the effect most pronounced for the ovaries.

Radiation exposure of the reproductive organs in pediatric females is a critical issue, given its potential association with long-term reproductive function and secondary cancer risk. The present findings quantitatively demonstrated that PA projection combined with copper filtration was effective in reducing not only breast and thyroid dose but also ovarian dose, thereby supporting dose optimization strategies in the long-term follow-up of adolescent idiopathic scoliosis. These results confirm that PA+Cu projection offers meaningful dose protection for reproductive organs, particularly the ovaries.

These organ-level findings were reflected in the total effective dose, which was reduced by approximately 50% under PA projection. With the addition of copper filtration, further decreases of 15–19% under AP projection and 5–6% under PA projection were observed, with the PA+Cu strategy yielding the greatest overall reduction. The effect was most pronounced in the 5-year-old model, indicating that superficial organs contributed strongly to determining the total effective dose.

Although effective dose is a pragmatic index, it remains essential for quantitatively discussing radiation risk in pediatric imaging. In the follow-up of adolescent idiopathic scoliosis, cumulative effective doses exceeding 20 mSv have been reported [14, 23]. In this study, the PA+Cu strategy reduced the effective dose by approximately 50%, suggesting substantial mitigation of this long-term risk. Moreover, the halving effect observed in this study further indicates significant potential for dose reduction. The findings also align with the principles advocated by the Image Gently campaign [29] and “International Commission on Radiological Protection Publication 121” [37], as well as the International Society on Scoliosis Orthopaedic and Rehabilitation Treatment (SOSORT) guidelines advocating PA projection [8], thereby reinforcing the clinical effectiveness of dose reduction strategies in pediatric patients.

The validity of these results was further supported by comparison with previous studies. The findings were in good agreement with previously reported thermoluminescent dosimeter (TLD) measurements and Monte Carlo analyses [6, 35, 36], and the validity of the model was confirmed as described in the “Results” section. In particular, the marked dose reductions for the breasts and thyroid with PA projection, as well as the additional reductions achieved with copper filtration, were consistent with trends reported in earlier studies [9, 14, 23]. Age-dependent variations observed in the lungs and deep-seated organs also fell within the ranges reported previously, further supporting the validity of the present model. Beyond validation, this study goes beyond many prior investigations that often focused on specific organs or imaging systems. Here, age-related changes in body thickness were incorporated, multiple organs were stratified from superficial to deep layers, and the comprehensive effects of copper filtration were quantitatively evaluated. This systematic and depth-stratified approach represents a unique contribution of the present study.

From a clinical perspective, the combination of PA projection and copper filtration effectively reduced superficial organ doses in pediatric full-spine radiography. The marked reductions observed in highly radiosensitive organs such as the breasts and thyroid directly translate into mitigating the cumulative risks associated with long-term follow-up of adolescent idiopathic scoliosis patients and provide evidence supporting the international guideline recommendations for PA projection. Conversely, increased doses were observed in posterior and deep-seated organs such as the kidneys and active bone marrow, highlighting the need for patient-specific protocol optimization tailored to organ location and individual characteristics rather than applying a uniform approach.

These findings have several clinical implications: (1) informing the design and revision of clinical protocols, incorporating PA projection and copper filtration as dose-reduction strategies; (2) enhancing radiation protection education, including patient explanation and informed consent that account for age- and body size–related risk differences; and (3) contributing to the development of future protection guidelines by providing optimization criteria specifically for pediatric imaging. Thus, beyond reaffirming prior knowledge, this study provides new foundational data for clinical implementation through comprehensive, age- and organ-stratified analyses.

With respect to copper filtration, transmitted photon counts decreased by 15–25%, and this reduction depended on tube voltage and body thickness. At higher tube voltages, the attenuation effect of copper filtration was relatively smaller, whereas in younger phantoms or under lower tube voltages, the beam-hardening effect was more pronounced. The additional use of a 0.1-mm copper filter reduced organ doses by approximately 20–40% and the effective dose by about 30% in our simulations, which is consistent with previous clinical findings by Minehiro et al. [7] in adolescent idiopathic scoliosis patients using the same filter thickness. Clinically, copper filtration is therefore a promising dose-reduction technique for younger children and low-kV imaging, although potential drawbacks such as decreased contrast and limited depiction of thick regions necessitate re-optimization of exposure parameters and image-processing strategies.

This study has several limitations. First, the evaluation was limited to female phantoms, and caution is warranted in generalizing the results to males or individuals with different body habitus. Second, Monte Carlo simulations were performed using relative doses per source, and conversion to absolute clinical doses (reflecting mAs or exposure time) would require additional correction. Third, diagnostic image quality was not quantitatively assessed; and therefore, the optimal balance between dose reduction and image quality could not be directly evaluated in this study. However, recent clinical and phantom studies in lumbar and cervical spine radiography have compared anteroposterior (AP) and posteroanterior (PA) projections, consistently demonstrating that PA imaging achieves substantial dose reduction while maintaining, and in some cases even improving, diagnostic image quality [38–41].

Furthermore, small absorbed doses were also observed in out-of-field regions, including the head and lower pelvis. These primarily reflect the contribution of scattered radiation, which, although low in magnitude, should be considered in the context of cumulative exposure from repeated examinations. Overall, while protocol optimization should consider organ location, age, and individual anatomy, PA projection with copper filtration may represent a balanced and clinically feasible approach for minimizing radiation risks in pediatric full-spine radiography.

## Conclusion

Using Monte Carlo simulations with 5-year-old, 10-year-old, and 15-year-old female phantoms, this study evaluated the effects of projection direction (AP vs. PA) and the addition of a 0.1-mm copper filter on organ and effective doses in pediatric full-spine radiography.

PA projection was associated with dose reductions of up to 90% in anterior radiosensitive organs such as the breasts and thyroid, with greater relative effects observed in younger age groups. In contrast, modest dose increases were noted in posterior deep-seated tissues such as the kidneys and active bone marrow, indicating trade-offs depending on organ location. Effective dose was reduced by approximately half with PA projection and further decreased by an additional 15–19% with copper filtration.

These observations are in line with the direction of radiation protection strategies advocated by the Image Gently campaign [29] and “International Commission on Radiological Protection Publication 121” [37] for optimization in repeated pediatric imaging. They provide useful data for considering the balance between organ protection and cumulative risk in full-spine radiography for adolescent idiopathic scoliosis patients. Furthermore, the reduction in effective dose with PA projection suggests potential clinical relevance in mitigating cumulative exposure during repeated follow-up examinations for scoliosis.

## Supplementary Information

Below is the link to the electronic supplementary material.

Supplementary Material 1 Rectangular irradiation fields for 5-, 10-, and 15-year-old phantoms. The fields were generated by defining a conical X-ray emission and applying a physical collimator. Visualization with the T-Track tally confirmed that the rectangular fields were accurately implemented in the Particle and Heavy Ion Transport code System (TIF 3.15 MB)

Supplementary Material 2 Normalized absorbed doses (mGy/source) and effective dose (mSv/source) for all 27 organs and tissues in 5-, 10-, and 15-year-old female phantoms under AP and PA projections, with and without copper filtration. All values are expressed per source. This table complements the representative 16 organs presented in Table 2 (37.0 KB)

Supplementary Material 3 Percentage reduction of absorbed organ doses and effective dose relative to AP projection without copper filtration (AP–Cu–) for all 27 organs and tissues in 5-, 10-, and 15-year-old female phantoms. The effects of PA projection and copper filtration are included. This table complements the representative 16 organs presented in Table 2 (37.1 KB)

## Data Availability

The datasets generated and analyzed during the current study are available from the corresponding author on reasonable request for non-commercial purposes.

## Abbreviations

AP: anteroposterior
PA: posteroanterior
Cu +: with copper filter applied
Cu–: without copper filter

## References

[1] Konieczny MR, Senyurt H, Krauspe R (2013) Epidemiology of adolescent idiopathic scoliosis. J Child Orthop 7(1):3–9. 10.1007/s11832-012-0457-4 24432052 10.1007/s11832-012-0457-4 PMC3566258

[2] Levy AR, Goldberg MS, Mayo NE, Hanley JA, Poitras B (1996) Radiation exposure to patients with idiopathic scoliosis. Spine 21:2680–2687

[3] Ronckers CM, Land CE, Miller JS et al (2010) Cancer mortality among women frequently exposed to radiographic exams for spinal disorders. Radiat Res 174:83–90. 10.1667/RR2022.1 20681802 10.1667/RR2022.1 PMC3982592

[4] Doody MM, Lonstein JE, Stovall M et al (2000) Breast cancer mortality after diagnostic radiography: findings from the U.S. scoliosis cohort study. Spine 25:2052–2063. https://journals.lww.com/spinejournal/fulltext/2000/08150/Breast_Cancer_Mortality_After_Diagnostic.9.aspx 10.1097/00007632-200008150-00009 10954636

[5] Deschênes S, Charron G, Beaudoin G et al (2010) Diagnostic imaging of spinal deformities: reducing patients’ radiation dose with a new slot-scanning X-ray imager. Spine 35:989–994. 10.1097/BRS.0b013e3181bdcaa4 20228703 10.1097/BRS.0b013e3181bdcaa4

[6] Pedersen PH Optimizing radiation dose and image quality in scoliosis imaging using EOS. PhD Thesis, Aalborg University (2019). Available at: https://vbn.aau.dk/files/549570631/PHD_Peter_Heide_Pedersen_E_pdf.pdf

[7] Minehiro K, Demura S, Ichikawa K et al (2019) Dose reduction protocol for full spine X-ray examination using copper filters in patients with adolescent idiopathic scoliosis. Spine 44:203–210. 10.1097/BRS.0000000000002787 30005046 10.1097/BRS.0000000000002787

[8] Negrini S, Donzelli S, Zaina F et al (2018) 2016 SOSORT guidelines: orthopaedic and rehabilitation treatment of idiopathic scoliosis during growth. Scoliosis Spinal Disord 13:3. 10.1186/s13013-017-0145-8 29435499 10.1186/s13013-017-0145-8 PMC5795289

[9] Almén A, Mattsson S (1995) Leitz W National survey on organ doses in pediatric radiography. Radiat Prot Dosimetry 57:373–376

[10] Guo H, Zhu Y, Qiu W et al (2013) Optimizing imaging quality and radiation dose by the age-dependent setting of tube voltage in pediatric chest digital radiography. Korean J Radiol 14:126–130 10.3348/kjr.2013.14.1.126 PMC3542296 23323043

[11] Alzen G, Benz-Bohm G (2011) Radiation protection in pediatric radiology. Dtsch Arztebl Int 108(24):407–414. 10.3238/arztebl.2011.0407 21776310 10.3238/arztebl.2011.0407 PMC3132617

[12] Seidenbusch M, Schneider K (2014) Conversion coefficients for determining organ doses in paediatric spine radiography. Pediatr Radiol 44(4):434–456. 10.1007/s00247-013-2853-4 24509648 10.1007/s00247-013-2853-4

[13] International Electrotechnical Commission (2009) Medical electrical equipment – Part 2-54: particular requirements for the basic safety and essential performance of X-ray equipment for radiography and radioscopy. IEC 60601-2-54. IEC, Geneva

[14] Ernst C, Buls N, Laumen A, Van Gompel G, Verhelle F, de Mey J (2018) Lowered dose full-spine radiography in pediatric patients with idiopathic scoliosis. Eur Spine J 27:2117–2124. 10.1007/s00586-018-5561-9 10.1007/s00586-018-5561-9 29589171

[15] International Commission on Radiological Protection (1982) Protection of the patient in diagnostic radiology. ICRP Publication 34. Ann ICRP 9(2–3):1–82 7171162

[16] Bushberg JT, Seibert JA, Leidholdt EM Jr et al (2012) The essential physics of medical imaging, 3rd ed. Lippincott Williams & Wilkins, Philadelphia

[17] Brosi P, Stuessi A, Verdun FR, Vock P, Wolf R (2011) Copper filtration in pediatric digital X-ray imaging: its impact on image quality and dose. Radiol Phys Technol 4(2):148–155. 10.1007/s12194-011-0115-4 21431385 10.1007/s12194-011-0115-4

[18] Staniszewska MA, Biegański T, Midel A, Barańska D (2000) Filters for dose reduction in conventional X-ray examinations of children. Radiat Prot Dosimetry 90(1–2):127–133. 10.1093/oxfordjournals.rpd.a033101

[19] Minehiro K, Matsumoto H, Yamada S et al (2019) Dose reduction protocol for full spine X-ray examination using copper filters in patients with adolescent idiopathic scoliosis. Spine (Phila Pa 1976) 44(3):203–210.10.1097/BRS.0000000000002787 10.1097/BRS.0000000000002787 30005046

[20] Gialousis G, Yiakoumakis EN, Makri TK et al (2008) Comparison of dose from radiological examination for scoliosis in children among two pediatric hospitals by Monte Carlo simulation. Health Phys. 94(5):471–478. 10.1097/01.HP.0000303105.91168.ea 18403968 10.1097/01.HP.0000303105.91168.ea

[21] Robinson SM, Esplen N (2020) Wells D monte carlo simulations of EBT3 film dose deposition for percentage depth dose (PDD) curve evaluation. J Appl Clin Med Phys 21:58–65. 10.1002/acm2.13078 33155768 10.1002/acm2.13078 PMC7769387

[22] Lee C, Lodwick D, Hurtado J et al (2010) The UF family of reference hybrid phantoms for computational radiation dosimetry. Phys Med Biol 55:339–363. 10.1088/0031-9155/55/2/002 20019401 10.1088/0031-9155/55/2/002 PMC2800036

[23] ICRP (2002) Basic anatomical and physiological data for use in radiological protection: reference values. ICRP Publication 89. Ann ICRP 32(3–4):1–277. 10.1016/S0146-6453(03)00002-2 14506981

[24] ICRP (2009) Adult reference computational phantoms. ICRP Publication 110. Ann ICRP 39(2):1–166. 10.1016/j.icrp.2009.09.001 10.1016/j.icrp.2009.09.001 19897132

[25] International Commission on Radiation Units and Measurements (1989) Tissue substitutes in radiation dosimetry and measurement. ICRU Report 44. ICRU, Bethesda

[26] International Commission on Radiation Units and Measurements (ICRU) (1992) Photon, electron, proton and neutron interaction data for body tissues. ICRU Report 46. ICRU, Bethesda

[27] Poludniowski GG, Evans P (2007) Calculation of X-ray spectra emerging from an X-ray tube. Part I. Electron penetration characteristics in X-ray targets. Med Phys 34(6):2164–2174. 10.1118/1.2734725 10.1118/1.2734725 17654919

[28] Poludniowski GG, Evans PM, Kavanagh A, Webb S (2009) SpekCalc: a program to calculate photon spectra from tungsten anode X-ray tubes. Phys Med Biol 54(19):N433–N438. 10.1088/0031-9155/54/19/N01 19724100 10.1088/0031-9155/54/19/N01

[29] Image Gently Alliance The Image Gently Campaign. Available at: https://www.imagegently.org (accessed Aug 2025)

[30] Sato T, Iwamoto Y, Hashimoto S et al (2024) Recent improvements of the particle and heavy ion transport code system – PHITS version 3.33. J Nucl Sci Technol. 61(2):127–135. 10.1080/00223131.2023.2275736

[31] ICRP (2007) The 2007 recommendations of the international commission on radiological protection. ICRP Publication 103. Ann ICRP 37(2–4):1–332. 10.1016/j.icrp.2007.10.003 10.1016/j.icrp.2007.10.003 18082557

[32] ICRP (2010) Conversion coefficients for use in radiological protection for external radiation exposures. ICRP Publication 116. Ann ICRP 40(2–5):1–257. 10.1016/j.icrp.2011.10.001 10.1016/j.icrp.2011.10.001 22386603

[33] ICRP (2002) Basic anatomical and physiological data for use in radiological protection: reference values. ICRP Publication 89. Ann ICRP 32(3–4):1–277. 10.1016/S0146-6453(03)00002-2 14506981

[34] ICRP (2020) Paediatric reference computational phantoms. ICRP Publication 143. Ann ICRP 49(1):1–83. 10.1177/0146645320914165 10.1177/0146645320915031 33000625

[35] Levy AR (1992) Projecting the lifetime risk of breast and thyroid cancer from exposure to diagnostic ionizing radiation for adolescent idiopathic scoliosis. MSc Thesis, McGill University, Montréal. Available at: https://escholarship.mcgill.ca/concern/theses/7p88ch732 10.1097/00004032-199406000-00002 8181937

[36] Gialousis G, Yiakoumakis EN, Makri TK et al (2008) Comparison of dose from radiological examination for scoliosis in children among two pediatric hospitals by Monte Carlo simulation. Health Phys 94:471–478. https://doi.org/10.1097/01.HP.0000303105.91168.ea 10.1097/01.HP.0000303105.91168.ea 18403968

[37] ICRP (2013) Radiological protection in paediatric diagnostic and interventional radiology. ICRP Publication 121. Ann ICRP 42:1–63. 10.1016/j.icrp.2012.10.001 10.1016/j.icrp.2012.10.001 23218172

[38] Davey E, England A (2015) AP versus PA positioning in lumbar spine computed radiography: image quality and individual organ doses. Radiography 21:188–196. 10.1016/j.radi.2014.11.003

[39] Alukić E, Skrk D, Mekiš N (2018) Comparison of anteroposterior and posteroanterior projection in lumbar spine radiography. Radiol Oncol 52:431–437. 10.2478/raon-2018-0021 10.2478/raon-2018-0021 PMC6287185 30511934

[40] Nemoto M, Chida K (2020) Reducing the breast cancer risk and radiation dose of radiography for scoliosis in children: a phantom study. Diagnostics 10:753. 10.3390/diagnostics10100753 32993028 10.3390/diagnostics10100753 PMC7600947

[41] Faulkner R, Lockwood P (2022) Could posterior–anterior projection cervical spine radiographs improve image quality and dose reduction? Radiography Open 8:42–50. Available from: https://journals.oslomet.no/index.php/radopen/article/view/5004

