# Supplementary figures and images for: Age-dependent evaluation of organ and effective doses in pediatric full-spine radiography: influence of anteroposterior and posteroanterior projection and copper filtration using Monte Carlo simulation
Source: Pediatr Radiol. 2025 Dec 11;56(3):603–17. doi: 10.1007/s00247-025-06452-7 (PMC12957027; doi:10.1007/s00247-025-06452-7)

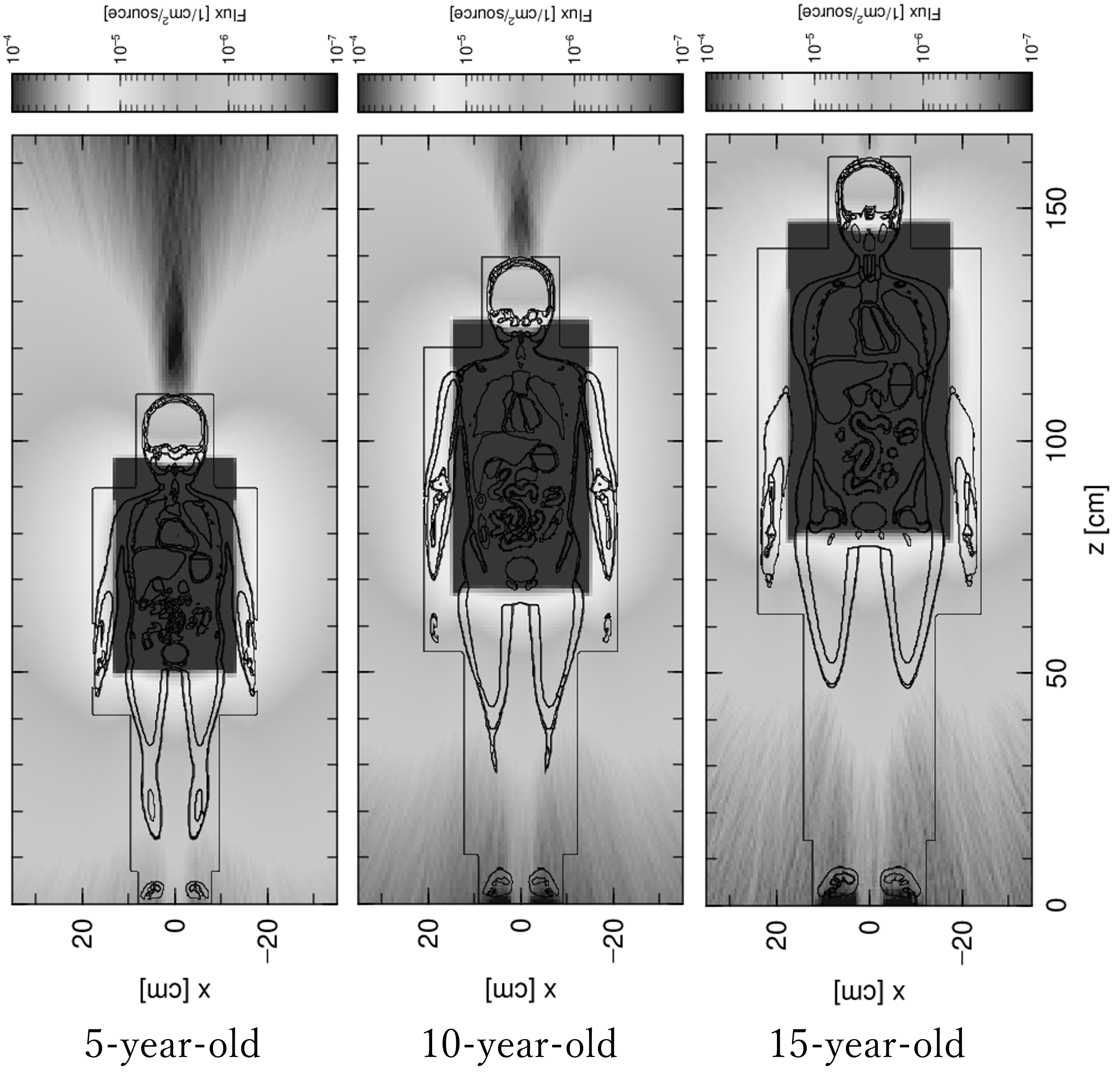

Supplement: Supplementary file 1 — Rectangular irradiation fields for 5-, 10-, and 15-year-old phantoms. The fields were generated by defining a conical X-ray emission and applying a physical collimator. Visualization with the T-Track tally confirmed that the rectangular fields were accurately implemented in the Particle and Heavy Ion Transport code System (TIF 3.15 MB) [file 247_2025_6452_MOESM1_ESM.tif]
