# Supplementary material for: Age-dependent evaluation of organ and effective doses in pediatric full-spine radiography: influence of anteroposterior and posteroanterior projection and copper filtration using Monte Carlo simulation
Source: Pediatr Radiol. 2025 Dec 11;56(3):603–17. doi: 10.1007/s00247-025-06452-7 (PMC12957027; doi:10.1007/s00247-025-06452-7)
Supplement: Supplementary file 2 — Normalized absorbed doses (mGy/source) and effective dose (mSv/source) for all 27 organs and tissues in 5-, 10-, and 15-year-old female phantoms under AP and PA projections, with and without copper filtration. All values are expressed per source. This table complements the representative 16 organs presented in Table 2 (37.0 KB) [file 247_2025_6452_MOESM2_ESM.docx]

**Supplementary Material 2**

Normalized absorbed doses (mGy/source) and effective dose (mSv/source) for all 27 organs and tissues in 5-, 10-, and 15-year-old female phantoms under AP and PA projections, with and without copper filtration. All values are expressed per source. This table complements the representative 16 organs presented in Table 2

|  | 5 years | | | | 10 years | | | | 15 years | | | |
| --- | --- | --- | --- | --- | --- | --- | --- | --- | --- | --- | --- | --- |
| Organ dose [mGy/source] | AP | PA | AP  Cu + | PA  Cu + | AP | PA | AP  Cu + | PA  Cu + | AP | PA | AP  Cu + | PA  Cu + |
| Active marrow | 0.28 | 0.40 | 0.25 | 0.36 | 0.22 | 0.38 | 0.20 | 0.34 | 0.26 | 0.35 | 0.23 | 0.32 |
| Breasts | 1.72 | 0.12 | 1.26 | 0.12 | 1.15 | 0.08 | 0.86 | 0.08 | 1.07 | 0.08 | 0.80 | 0.07 |
| Colon | 1.42 | 0.51 | 1.12 | 0.46 | 0.98 | 0.26 | 0.81 | 0.25 | 0.97 | 0.19 | 0.78 | 0.15 |
| Lungs | 1.05 | 1.05 | 0.87 | 0.90 | 0.61 | 0.64 | 0.54 | 0.58 | 0.67 | 0.47 | 0.56 | 0.36 |
| Stomach | 1.01 | 0.58 | 0.88 | 0.53 | 0.71 | 0.33 | 0.63 | 0.32 | 0.76 | 0.28 | 0.64 | 0.22 |
| Ovaries | 0.90 | 0.58 | 0.79 | 0.54 | 0.45 | 0.37 | 0.42 | 0.37 | 0.42 | 0.23 | 0.39 | 0.18 |
| Bladder(Uri.) | 1.49 | 0.43 | 1.18 | 0.40 | 0.70 | 0.30 | 0.62 | 0.28 | 0.51 | 0.17 | 0.45 | 0.14 |
| Esophagus | 0.76 | 0.60 | 0.67 | 0.57 | 0.44 | 0.41 | 0.40 | 0.40 | 0.41 | 0.36 | 0.36 | 0.29 |
| Liver | 0.95 | 0.66 | 0.83 | 0.59 | 0.63 | 0.45 | 0.57 | 0.41 | 0.73 | 0.30 | 0.62 | 0.24 |
| Thyroid | 2.23 | 0.47 | 1.65 | 0.45 | 1.53 | 0.26 | 1.17 | 0.25 | 1.40 | 0.24 | 1.06 | 0.18 |
| Bone surface | 0.43 | 0.65 | 0.39 | 0.58 | 0.25 | 0.44 | 0.24 | 0.41 | 0.26 | 0.33 | 0.23 | 0.30 |
| Brain | 0.01 | 0.02 | 0.02 | 0.02 | 0.01 | 0.02 | 0.02 | 0.02 | 0.01 | 0.02 | 0.01 | 0.02 |
| Salivary blands | 0.54 | 0.65 | 0.47 | 0.54 | 0.50 | 0.55 | 0.44 | 0.46 | 0.35 | 0.43 | 0.30 | 0.35 |
| Skin | 0.54 | 0.53 | 0.38 | 0.38 | 0.33 | 0.33 | 0.25 | 0.25 | 0.33 | 0.32 | 0.24 | 0.23 |
| Reminder | 0.72 | 0.70 | 0.62 | 0.60 | 0.47 | 0.46 | 0.43 | 0.42 | 0.46 | 0.40 | 0.39 | 0.36 |
| Adrenals | 0.27 | 1.28 | 0.26 | 1.08 | 0.17 | 0.88 | 0.18 | 0.79 | 0.19 | 0.70 | 0.18 | 0.60 |
| ET regions | 0.72 | 0.31 | 0.58 | 0.28 | 0.80 | 0.25 | 0.63 | 0.24 | 0.90 | 0.20 | 0.69 | 0.19 |
| Gall_bladder | 0.78 | 0.60 | 0.73 | 0.53 | 0.56 | 0.34 | 0.54 | 0.32 | 0.53 | 0.26 | 0.48 | 0.25 |
| Heart | 1.25 | 0.49 | 1.05 | 0.46 | 0.76 | 0.30 | 0.68 | 0.29 | 0.79 | 0.23 | 0.67 | 0.22 |
| Kidneys | 0.40 | 1.34 | 0.39 | 1.13 | 0.20 | 1.01 | 0.20 | 0.87 | 0.17 | 1.00 | 0.16 | 0.84 |
| Lympa._nodes | 0.57 | 0.59 | 0.45 | 0.47 | 0.36 | 0.37 | 0.30 | 0.31 | 0.29 | 0.34 | 0.24 | 0.28 |
| Muscle | 0.51 | 0.67 | 0.43 | 0.55 | 0.38 | 0.41 | 0.33 | 0.35 | 0.23 | 0.35 | 0.20 | 0.29 |
| Oral_mucosa | 0.41 | 0.21 | 0.35 | 0.21 | 0.33 | 0.17 | 0.33 | 0.17 | 0.48 | 0.20 | 0.41 | 0.18 |
| Pancreas | 0.65 | 0.78 | 0.59 | 0.71 | 0.42 | 0.44 | 0.40 | 0.42 | 0.34 | 0.47 | 0.31 | 0.43 |
| Uterus | 0.67 | 0.54 | 0.59 | 0.49 | 0.37 | 0.36 | 0.34 | 0.34 | 0.27 | 0.26 | 0.25 | 0.24 |
| Small_int | 1.21 | 0.47 | 1.02 | 0.44 | 0.78 | 0.26 | 0.69 | 0.26 | 0.65 | 0.27 | 0.56 | 0.25 |
| Spleen | 0.46 | 1.37 | 0.43 | 1.14 | 0.29 | 0.92 | 0.28 | 0.79 | 0.37 | 0.76 | 0.34 | 0.64 |
| Thymus | 1.46 | 0.40 | 1.19 | 0.37 | 0.70 | 0.29 | 0.64 | 0.28 | 0.74 | 0.21 | 0.62 | 0.20 |
| Effective dose | 1.05 | 0.55 | 0.85 | 0.50 | 0.67 | 0.36 | 0.57 | 0.33 | 0.67 | 0.28 | 0.55 | 0.26 |
