# Supplementary material for: Age-dependent evaluation of organ and effective doses in pediatric full-spine radiography: influence of anteroposterior and posteroanterior projection and copper filtration using Monte Carlo simulation
Source: Pediatr Radiol. 2025 Dec 11;56(3):603–17. doi: 10.1007/s00247-025-06452-7 (PMC12957027; doi:10.1007/s00247-025-06452-7)
Supplement: Supplementary file 3 — Percentage reduction of absorbed organ doses and effective dose relative to AP projection without copper filtration (AP–Cu–) for all 27 organs and tissues in 5-, 10-, and 15-year-old female phantoms. The effects of PA projection and copper filtration are included. This table complements the representative 16 organs presented in Table 2 (37.1 KB) [file 247_2025_6452_MOESM3_ESM.docx]

**Supplementary Material 3**

Percentage reduction of absorbed organ doses and effective dose relative to AP projection without copper filtration (AP–Cu–) for all 27 organs and tissues in 5-, 10-, and 15-year-old female phantoms. The effects of PA projection and copper filtration are included. This table complements the representative 16 organs presented in Table 3

|  | 5 years | | | | 10 years | | | | 15 years | | | | |
| --- | --- | --- | --- | --- | --- | --- | --- | --- | --- | --- | --- | --- | --- |
| Organ dose [mGy/source] | AP | PA | AP  Cu + | PA  Cu + | AP | PA | AP  Cu + | PA  Cu + | AP | PA | AP  Cu + | PA  Cu + |  |
| Active marrow | - | 46.7 | -11.1 | 29.0 | - | 74.5 | -7.2 | 58.8 | - | 36.2 | -10.9 | 22.1 |  |
| Breasts | - | -92.9 | -26.7 | -92.9 | - | -93.0 | -25.1 | -92.7 | - | -92.8 | -25.4 | -93.1 |  |
| Colon | - | -64.4 | -20.7 | -67.8 | - | -73.7 | -17.2 | -74.7 | - | -80.7 | -19.1 | -84.6 |  |
| Lungs | - | 0.0 | -17.2 | -14.7 | - | 5.0 | -11.0 | -5.2 | - | -30.1 | -16.3 | -46.7 |  |
| Stomach | - | -42.4 | -12.9 | -47.5 | - | -52.8 | -10.2 | -54.6 | - | -63.8 | -15.3 | -71.2 |  |
| Ovaries | - | -36.0 | -12.4 | -39.7 | - | -18.6 | -6.9 | -18.6 | - | -46.9 | -8.4 | -58.6 |  |
| Bladder(Uri.) | - | -71.2 | -20.5 | -73.2 | - | -57.4 | -11.2 | -59.3 | - | -67.0 | -12.1 | -73.2 |  |
| Esophagus | - | -21.0 | -11.8 | -25.0 | - | -7.0 | -9.5 | -9.1 | - | -13.1 | -11.7 | -29.5 |  |
| Liver | - | -30.2 | -12.9 | -37.3 | - | -29.5 | -10.1 | -35.0 | - | -58.4 | -14.4 | -67.2 |  |
| Thyroid | - | -78.9 | -26.1 | -79.8 | - | -82.8 | -23.5 | -83.4 | - | -82.9 | -24.1 | -86.8 |  |
| Bone surface | - | 50.9 | -9.1 | 35.1 | - | 78.4 | -4.9 | 65.3 | - | 29.2 | -9.7 | 17.0 |  |
| Brain | - | 44.1 | 1.8 | 43.0 | - | 51.6 | 4.6 | 50.3 | - | 26.1 | -3.1 | 20.8 |  |
| Salivary blands | - | 20.0 | -13.1 | -1.6 | - | 8.8 | -13.6 | -9.2 | - | 21.3 | -14.1 | 0.5 |  |
| Skin | - | -1.5 | -28.9 | -29.9 | - | -0.7 | -25.6 | -26.0 | - | -2.0 | -27.6 | -29.0 |  |
| Reminder | - | -3.2 | -13.9 | -15.9 | - | -2.4 | -9.6 | -11.4 | - | -11.6 | -14.2 | -22.5 |  |
| Adrenals | - | 374.0 | -2.9 | 300.1 | - | 402.7 | 2.0 | 350.2 | - | 273.3 | -4.8 | 219.7 |  |
| ET regions | - | -57.3 | -20.1 | -61.4 | - | -69.3 | -21.4 | -70.6 | - | -77.5 | -23.7 | -78.6 |  |
| Gall_bladder | - | -23.1 | -6.5 | -31.5 | - | -39.8 | -4.2 | -43.1 | - | -50.2 | -8.5 | -53.0 |  |
| Heart | - | -60.5 | -15.9 | -63.2 | - | -61.0 | -11.0 | -61.9 | - | -71.1 | -14.9 | -72.7 |  |
| Kidneys | - | 231.5 | -4.5 | 179.6 | - | 410.6 | -0.7 | 340.6 | - | 506.6 | -4.8 | 409.2 |  |
| Lympa._nodes | - | 4.4 | -20.2 | -16.7 | - | 2.0 | -16.1 | -14.4 | - | 15.9 | -18.4 | -5.9 |  |
| Muscle | - | 31.4 | -16.2 | 6.5 | - | 6.4 | -13.9 | -8.7 | - | 54.2 | -13.4 | 28.8 |  |
| Oral_mucosa | - | -47.6 | -13.8 | -49.3 | - | -48.8 | -0.5 | -48.3 | - | -58.4 | -14.6 | -62.0 |  |
| Pancreas | - | 19.8 | -9.1 | 9.7 | - | 6.0 | -2.8 | 0.8 | - | 39.2 | -7.7 | 25.6 |  |
| Uterus | - | -19.6 | -12.1 | -26.7 | - | -1.7 | -8.1 | -7.0 | - | -5.7 | -9.0 | -11.2 |  |
| Small_int | - | -61.4 | -15.5 | -63.6 | - | -66.5 | -11.2 | -67.0 | - | -58.6 | -13.4 | -61.2 |  |
| Spleen | - | 201.0 | -6.3 | 150.6 | - | 219.3 | -3.3 | 175.3 | - | 103.4 | -10.0 | 72.3 |  |
| Thymus | - | -72.7 | -18.7 | -74.5 | - | -59.1 | -8.4 | -59.7 | - | -71.6 | -16.2 | -73.4 |  |
| Effective dose | - | -47.1 | -19.0 | -52.7 | - | -47.1 | -15.5 | -50.6 | - | -57.6 | -17.9 | -61.6 |  |
